# Syntactic and affective prosody recognition: Schizophrenia vs. Autism spectrum disorders

**DOI:** 10.1371/journal.pone.0292325

**Published:** 2023-10-05

**Authors:** Maria Martzoukou, Dimitrios Papadopoulos, Mary H. Kosmidis

**Affiliations:** Lab of Cognitive Neuroscience, School of Psychology, Aristotle University of Thessaloniki, Thessaloniki, Greece; Catholic University of the Sacred Heart: Universita Cattolica del Sacro Cuore, ITALY

## Abstract

Patients with a recent diagnosis of schizophrenia and individuals receiving a diagnosis of autism spectrum disorder without accompanying intellectual impairment (ASD w/o intellectual impairment) during their adulthood share several clinical characteristics. Exploring under-investigated aspects of these two clinical conditions may shed light on their possible connection and facilitate differential diagnosis at very early stages. To this end, we explored the ability of 15 adults with a recent diagnosis of schizophrenia, 15 individuals diagnosed with ASD w/o intellectual impairment as adults, and 15 healthy adults to resolve sentence ambiguities with the use of syntactic prosody, and to decode happiness, anger, sadness, surprise, fear, and neutrality based on affective prosody. Results revealed intact perception of syntactic prosody in adults with schizophrenia, but impaired affective prosody recognition, which could be attributed, however, to emotion processing difficulties overall. On the other hand, individuals with ASD w/o intellectual impairment were impaired on prosody comprehension *per se*, as evidenced in the most challenging conditions, namely the subject-reading condition and the emotion of surprise. The differences in prosody comprehension ability between the two clinical conditions may serve as an indicator, among other signs, during the diagnostic evaluation.

## 1. Introduction

Schizophrenia is a neuropsychiatric disorder characterized by distortions in thinking, perception, emotions, language, sense of self, and behavior [1]. It usually appears in late adolescence or early adulthood, and it develops earlier in men than in women [1]. The prevalence of the disorder is around 0.5% globally, and it affects women and men equally (e.g., [1]).

For an individual to be diagnosed with schizophrenia, one or more of the following symptoms must be present: delusional beliefs, illusions, disorganized thinking/speech, severely disorganized or abnormal motor behavior (catatonia), and negative symptoms, namely reduced expression of emotions, resignation, apathy, anhedonia, and reduced interpersonal functioning [2]. The causes of schizophrenia are not clear, but several factors have been found to be related to the disorder, such as heredity, neurotransmitters (particularly dopamine and serotonin), and structural brain differences [3]. Other factors, however, such as perinatal complications, drug abuse, and stressful situations, appear to worsen the symptoms of schizophrenia or increase the risk of development in someone already vulnerable to the disorder [3].

The term “schizophrenia” was first coined by the psychiatrist Eugen Bleuler to denote the “splitting of the mind”, namely the splitting of the psychic functions [4]. Interestingly, Bleuler, in his description of schizophrenia in 1911 [5], was also the first who used the term “autism” to describe a state of complete insulation from reality in people with schizophrenia [4]. Later, both Kanner [6] and Asperger [7] used this term, referring to “early infantile autism” and “autistic psychopaths”, respectively, to describe the conditions of two groups of boys, characterized by qualitative impairments in social interaction and communication and restricted patterns of interest and behaviors. Modern diagnostic terminology has placed this neurodevelopmental disorder under the umbrella of Autism Spectrum Disorder (ASD) with persistent deficiencies in social communication and interaction, and limited and repetitive patterns of behavior, interests and activities to be the main diagnostic criteria (DSM-5; [2]).

Given that individuals with this syndrome may or may not have intellectual disability and that these two subgroups are substantially different from each other, in the present study we focus on those without accompanying intellectual impairment (ASD w/o intellectual impairment). ASD w/o intellectual impairment is present from birth, but usually becomes noticeable at the age of 5 or 6 years old [8]. The prevalence of the disorder ranges from 0.67 to 48 cases out of 10.000 births and the female to male ratio is 1:4 [8] (the range of prevalence cited is specific to Brick Township, New Jersey, and, thus, it may differ in other locations). The causes are not clear, but factors, such as heredity, prenatal substance abuse, perinatal complications, and impaired brain areas or problematic neural connections have been suggested [9,10] (for a recent review on neural networks in schizophrenia and ASD see [11]).

Some commonalities exist between the two conditions. One such commonality is that both adults with schizophrenia and those with ASD w/o intellectual impairment face difficulties in social interaction [12,13]. In the former group, however, this reflects an intentional social withdrawal or a social withdrawal due to stigma [14], whereas in the latter group, inability to understand and use the rules of conversation (e.g., back-and-forth conversation) [12,13]. Moreover, the general anhedonia and resignation observed in adults with schizophrenia might be confused with the limited interests observed in individuals with ASD w/o intellectual impairment [12,13]. Individuals of both disorders may also exhibit persecutory ideas or delusions as a result of their perception of the social environment as aggressive and indifferent [12,13]. The origin of these characteristics varies by condition, namely it could be either an aftereffect of hallucinations and misinterpretation of reality (schizophrenia) or an aftereffect of the individual’s inability to understand social interactions (ASD) [12,13]. Other deficits, such as poor theory of mind, inability to interpret facts, limited working memory, slow data processing speed, and lack of expressiveness have been reported in several studies for both groups [15–19].

Recent studies demonstrate that the association between schizophrenia and ASD w/o intellectual impairment is still under investigation [20–22] and suggest that the clinical overlap could lead to misdiagnosis and, consequently, to a delay in providing suitable interventions and treatments. Another major challenge in the differential diagnosis is that both disorders are characterized by a great heterogeneity, and, thus, symptoms can vary significantly between different individuals [13]. Therefore, the evaluation of other behavioral characteristics might be a valuable supplement both to research regarding the differences and the similarities between the two disorders and to the demanding clinical differentiation between ASD w/o intellectual impairment and early stages of schizophrenia in adults. In this respect, we compared the ability of individuals diagnosed with ASD w/o intellectual impairment in their adulthood to that of adults with a recent diagnosis of schizophrenia to comprehend affective and syntactic prosody and whether such an evaluation could serve as a supplementary test in the diagnostic process.

Regarding affective prosody comprehension, previous research conducted with adults with mild autism resulted in contradictory results, as some studies reported impaired recognition of affective prosody [23–30], while others reported similar performance to that of typically developed, healthy adults [31–36] (for a recent review and a meta-analysis see also [37]). Similarly, the sparse research conducted on patients with schizophrenia have not clarified whether the impairment is generalized or focused on specific emotions [38–43] (for a recent review and a meta-analysis see also [44]). These differences have been attributed to variations in research procedures, emotions explored, and voices/manners in which the emotions were delivered.

In comparison to affective prosody, little is known about the comprehension of syntactic prosody in both medical conditions. In particular, two studies of adults with ASD investigating the perception of prosody to resolve syntactic ambiguity [28,45] yielded contradictory results, with authors [28] reporting worse performance of participants with ASD compared to controls and Paul et al. [45] reporting similar performance in both groups. As for patients with schizophrenia, sentence phrasing based on prosody has been tested by Matsumoto et al. [46] and Rabagliati et al. [47]. The difference between the performance of patients with schizophrenia and that of the control groups did not reach significance in either of the two studies (for non-affective prosody in patients with schizophrenia see also Lucarini et al. [48]). The small number of studies on this issue, however, indicates that more research is required. Furthermore, to our knowledge, there is no study comparing the performance of both adults with ASD w/o intellectual impairment and adults with schizophrenia on the same prosody tasks. Thus, a study including both clinical groups using the same tasks recorded by the same actor/actress, following the same procedure, and exploring the same emotions or syntactically ambiguous sentences is clearly needed. Our goal is to fill this gap in the literature.

## 2. Method

### 2.1. Participants

Fifteen patients with a recent diagnosis of schizophrenia participated in the study between January 2021 and December 2022. Moreover, 15 adults who have been diagnosed in adulthood as individuals with ASD w/o intellectual impairment, and 15 healthy adults took part in the present study (Table 1).

**Table 1:** Demographic characteristics of each group; means and SDs in parentheses.

Groups	Sex	Age in years (SD)	Years of education (SD)
CG	4F/11M	28.8 (9.9)	14.8 (1.9)
ScG	4F/11M	29.9 (10.7)	14.0 (1.5)
ASD	4F/11M	30.5 (10.9)	14.0 (1.5)

CG = Control group, ScG = Group with schizophrenia, ASD = Autism Spectrum Disorder without intellectual impairment, F = female, M = Male.

More specifically, adults with schizophrenia were recruited from the acute ward of a public psychiatric hospital and had received a diagnosis of schizophrenia between 9–30 months prior to participation in the study. All patients were diagnosed, at the time of recruitment, according to DSM-V criteria [2]. The diagnosis was confirmed with the Greek version [49] of the Mini International Neuropsychiatric Interview [50]. The exclusion criteria were (a) a diagnosis more than 30 months, (b) being bilingual or not a native speaker or Greek, (c) having any sensory, hearing, learning or cognitive impairment and, (d) having any other neurobehavioral or genetic disorder. All patients with schizophrenia were receiving atypical antipsychotic medication (i.e., Abilify, Risperdal) at the time of the study.

Individuals with ASD w/o intellectual impairment were recruited from a larger cohort of individuals (N = 20) who had taken part in a previous investigation [28]. For the present study, we selected among the original participants with ASD w/o intellectual impairment, those who matched individual patients with schizophrenia by gender, age, and years of education (the latter two by approximation). All 15 individuals sought a diagnosis after watching a TV program in which the president of the “Association of Greek Autistic, Asperger’s and HFA Adults” described the characteristics of the syndrome. These adults were later diagnosed as having ASD w/o intellectual impairment (in particular Asperger’s syndrome) according to the DSM-IV [51] and the International Classification of Diseases-10 (ICD-10; [52]) criteria by a certified psychiatrist experienced in autistic spectrum disorders. None had a history of speech delay, and all had normal or above normal intelligence according to the Greek version of the Wechsler Adult Intelligence Scale (WAIS-IV, [53]; for Greek [54]). Non-native speakers of Greek or bilingual individuals were excluded, as well as individuals with sensory, hearing, or learning impairments. Additionally, adults with ASD w/o intellectual impairment who had been diagnosed with any other neurobehavioral or genetic disorder were also excluded. Lastly, a group of 15 healthy adults, matched to individuals of the two former groups based on sex, age, and education, was used as the control group. Controls were recruited from the community and took part voluntarily. All participants were monolingual, native speakers of Greek with 12 to 18 years of education. None of them had any other neurological or developmental disorder or any physical illness that may have affected their cognitive condition. Moreover, none had suffered a head injury followed by loss of consciousness or had a history of alcohol or drug abuse. Additionally, healthy participants had no history of a psychiatric disorder or treatment, or a family history of psychosis or autism. All data were collected by the first and the second author.

Before the main tests were administered, participants with schizophrenia (and controls) were assessed with a battery of cognitive tasks, as possible predictors of their performance on the main tasks. In particular, we assessed attention and mental processing speed, as well as shifting ability in patients with schizophrenia with the use of the Trail Making Test (TMT). In Part A (TMT-A) of this test, patients had to connect, by drawing lines on a sheet of paper, a set of 25 circled numbers in a numerical sequence as fast as possible. In Part B (TMT-B), participants were instructed to connect circled numbers (from 1 to 13) and letters (from A to M) in an alternating numeric and alphabetic sequence, as quickly as possible (for Greek norms see [55]).

Moreover, short-term verbal memory was assessed with the use of a 10-word list [56], which was orally presented to participants and repeated four times. After every oral presentation of the words, participants were asked to recall as many as they could. The dependent variable was the total number of words recalled across the five trials (see Table 2).

**Table 2:** Mean accuracy (standard deviations) in percentages for each group on the recognition of each emotion.

Emotion	ScG	ASD	CG
Happiness	69 (46)	83 (38)	92 (27)
Sadness	61 (49)	84 (37)	93 (25)
Fear	73 (45)	80 (40)	91 (29)
Surprise	67 (48)	52 (50)	92 (27)
Neutrality	89 (31)	89 (31)	96 (20)
Anger	80 (40)	92 (27)	91 (29)

Note: ScG = Group with schizophrenia, ASD = Autism Spectrum Disorder w/o intellectual impairment, CG = Control group.

Lastly, we evaluated global functioning of all participants in both clinical groups according to the Global Assessment of Functioning (GAF; [51]). GAF is used to evaluate how seriously a disorder has affected one’s psychological, social, and professional functioning. The GAF score ranges from 0 to 100, where higher scores indicate better functioning.

The present work was approved by the Committee for Bioethics and Ethics of the School of Medicine of the Aristotle University of Thessaloniki, Greece, (No. 1330). The committee examined the scientific protocol for research involving humans, as well as the participants’ information and consent form, before granting approval. Written consent was obtained from all participants of the study after having been informed, in both written and oral form, about the nature of the study.

### 2.2. Materials

#### 2.2.1. Affective prosody task

Participants’ ability to decode the speaker’s emotion via the voice was assessed with the use of the Affective Prosody Task (APT [57]; see also [28,38,58,59]). APT consists of a total of 30 audio-recorded sentences, produced by a male actor. Sentences have emotionally neutral content (e.g., *The furniture is made of wood*), but they are produced with prosody revealing one of six emotions: neutrality, happiness, sadness, surprise, fear, and anger. Each emotion is presented in five trials with different sentence contents each time.

#### 2.2.2. Syntactic prosody task

Comprehension of syntactic prosody was examined with the use of subject/object ambiguous onsets of sentences. In particular, participants listened to 12 pre-recorded sentence -onsets containing a noun phrase (NP) which could either be the subject of the main sentence (subject-reading) or the object of the subordinate clause (object reading).

For example, the sentence-onsets (1a) and (1b) are temporarily ambiguous in that the NP *ta* ’*γramata* could either be the subject of the main verb or the object of the subordinate one (’δjavaze).

#### 1. a. Subject-reading

ka’θos ’δjavaze / ta ’γramata.

while was-reading the letters_NEUT.PL.NOM/ACC.

While (s)he was reading the letters….

#### b. Object-reading

ka’θos ’δjavaze ta ’γramata /….

while was-reading the letters_NEUT.PL.NOM/ACC.

While (s)he was reading the letters….

The temporal ambiguity results from to the fact that, although Greek nouns and articles are inflected for case, number and gender, the nominative and accusative forms overlap in the neuter gender [60]. Thus, a noun in the neuter gender could act either as the subject of the main clause (nominative case) or as the object of the subordinate clause (accusative case). The ambiguity of the sentence-onset in (1) is resolved by means of prosody. In particular, depending on the location of the prosodic boundary (depicted with the use of slashes), before or after the ambiguous NP, the NP serves as subject or object respectively.

The continuations provided by the listeners can denote whether they took prosodic cues into consideration. In particular, Greek verbs are inflected for person and number and, thus, an “-*e*” ending of a verb indicates the third person singular in past tense, whereas the ending “-an” indicates the third person plural in past tense. Therefore, a correct completion in (1a) would be one starting with a third plural verb (and not containing any other overt subject) indicating that the given DP was taken as the subject of the main verb (e.g., ’arhis**an** na θo’lonun–they started to blur). On the contrary, continuations starting with a verb in the third singular (with or without an overt subject) [e.g., za’listik**e**—(s/he) felt dizzy] or in any other person accompanied by an overt subject, either a DP or a pronominal, [e.g., ta pe’δja ’irθan–the children came] are in line with the onsets produced with prosody in favour of the object reading condition.

In the study there were also 24 unambiguous sentence-onset fragments, 12 in favour of the subject reading and 12 in favour of the object reading, which were used as fillers. The unambiguous subject reading sentences contained an intransitive embedded verb, making the object reading unavailable, while the unambiguous object reading sentences contained a (optionally) transitive verb followed by a NP, marked for the accusative case, making the subject reading impossible. We included fillers that had a similar structure to the experimental items for two main reasons. Firstly, we wanted to investigate if participants encountered any challenges with the structures used in our methodology. Secondly, we aimed to determine whether the combination of both morphosyntax and prosody aided listeners in accurately processing the sentence fragments.

### 2.3. Procedure

All participants were tested individually in a quiet room either at their place of residence (participants with ASD w/o intellectual impairment and controls) or at the hospital (participants with schizophrenia).

#### 2.3.1. Affective prosody task

As for the administration of APT, the sentences were orally presented to participants in a pseudo-randomized order with a 9-second pause between them. Participants had to choose and orally indicate one of the six emotions written on a piece of paper placed in front of them. The procedure lasted approximately 7 min per participant. A training trial in which participants heard the same sentence produced with each of the six emotions preceded the main experiment, for participants to regulate the desired intensity of the sound and to become familiarized with the task.

#### 2.3.2. Syntactic prosody task

Regarding the syntactic prosody task, participants were informed that they were going to listen to sentence-onsets, which they would have to complete by giving a reasonable continuation. The experimental items and the fillers were presented to participants in pseudo-randomized order. All participants listened to both lists. The experiment was divided into two sessions, one for each list, which participants undertook with a period of at least one week between them, in order for possible memory effects to be avoided.

### 2.4. Hypotheses

#### 2.4.1. Affective prosody task

Participants with schizophrenia (a1) are expected to demonstrate difficulties with affective prosody decoding, particularly with the recognition of negative emotions; this hypothesis is based on previous use of the same affective prosody task [38], and others [39–41]. According to previous findings of our group with ASD w/o intellectual impairment [28], we expect our participants (a2) to encounter difficulties with the recognition of surprise.

#### 2.4.2. Syntactic prosody task

As for the syntactic prosody task, the group with schizophrenia (b1) is expected to perform similarly to the control group [46,47]. Based on previous findings of the group with ASD w/o intellectual impairment taken part in the present study, (b2) we expect it to perform worse compared to the controls in the subject reading condition of the experimental items.

Moreover, (c) we anticipate a poor performance of the participants with schizophrenia on attention, mental processing speed, shifting ability, and verbal short-term memory, as measured with the use of the corresponding tests (for their poor cognitive abilities see also [15–19]), to affect their performance on both APT and syntactic prosody tasks. Similarly, (d) the two clinical groups’ performance on GAF is expected to be related to their ability to recognise affective and syntactic prosody. In particular, their limited social interaction [13,14] is expected to be associated to their poor social functioning and both of them to have negatively impacted their ability to understand communication cues such as prosody. Lastly, (e) a connection between the ability to recognise affective and syntactic prosody is expected to be found for both clinical groups (see also [28]).

### 2.5. Statistical analyses

Normality was assessed using the Shapiro–Wilk test per group and per condition. All data were found to be non-normally distributed. Therefore, between-subjects analyses were performed with Kruskal-Wallis tests. Differences between the controls and each of the clinical groups were estimated via Mann-Whitney U tests. For the effect sizes, rank biserial correlations (r) were used.

Pearson’s correlations were used to evaluate the relationship between participants’ performance on TMT-A, TMT-B, 10-word list and GAF on the one hand, and their performance on APT and the syntactic prosody task on the other, for all groups, as variables met the assumptions of normality determined by the Shapiro–Wilk test for Bivariate Normality. Similarly, Pearson’s correlations were used to explore the relationship between APT and syntactic prosody task performance for each clinical group separately.

## 3. Results

### 3.1. Affective prosody task

In order to explore the three groups’ performance on each of the emotions explored by APT, Kruskal-Wallis was conducted. The results revealed a group difference on the recognition of the emotions of happiness (χ^2^ = 12.765, p = .002, η^2^ = .057), sadness (χ^2^ = 24.865, p < .001, η^2^ = .111), fear (χ^2^ = 7.519, p = .023, η^2^ = .034), and surprise (χ^2^ = 29.243, p < .001, η^2^ = .131), whereas no group differences were found on the recognition of neutrality (χ^2^ = 2.862, p = .239, η^2^ = .013). Lastly, a marginally significant effect was presented on the recognition of anger (χ^2^ = 5.929, p = .052, η^2^ = .026).

Further analysis revealed that the group of patients with schizophrenia performed significantly worse than the control group in the recognition of all emotions apart from neutrality and anger (happiness: U = 2175.000, p < .001, r = .227; sadness: U = 1912.000, p < .001, r = .320; fear: U = 2325.000, p = .006, r = .173; surprise: U = 2100.000, p < .001, r = .253; neutrality: U = 2625.000, p = .120, r = .067; anger: U = 2512.500, p = .066, r = .107). The performance of the ASD w/o intellectual impairment group was significantly lower than that of the control group only on the recognition of surprise (happiness: U = 2550.000, p = .087, r = .093; sadness: U = 2550.000, p = .073, r = .093; fear: U = 2512.000, p = .066, r = .107; surprise: U = 1687.500, p < .001, r = .400; neutrality: U = 2625.000, p = .120, r = .067; anger: U = 2850.000, p = .775, r = .013) (see also Table 2).

As for incorrect responses, the results revealed some confusion between the emotions of fear and sadness, in that fear was perceived as sadness and sadness as fear in all three groups. A similar confusion was observed between happiness and surprise, in that almost all incorrect responses given by participants of the three groups on happiness were in favour of the emotion of surprise. The opposite, however, did not occur in either group (that is, surprise was not confused with happiness). Interestingly, in most cases individuals with schizophrenia and healthy adults mistook surprise as neutrality, whereas individuals with ASD w/o intellectual impairment perceived surprise as expressing fear (see Table 3). As for anger and neutrality, they led to very few erroneous answers, thus no clear conclusions can be extracted.

**Table 3:** Distribution of incorrect responses (%) of each of the three groups in each of the six target emotions (horizontal row). [The most common errors of each group on each of the targeted emotions are indicated in bold font].

	Happiness			Surprise			Fear			Sadness			Neutrality			Anger		
	ScG	ASD	CG	ScG	ASD	CG	ScG	ASD	CG	ScG	ASD	CG	ScG	ASD	CG	ScG	ASD	CG
**Happiness**				21	15								**50**			10	25	
**Sadness**				4	19	25	**50**	**85**	**100**				25		**100**			
**Fear**				11	**52**					**76**	**80**	**95**	25	12		10	25	
**Surprise**	**94**	**89**	**100**				14	5		8				26		30	**50**	20
**Neutrality**	6	11		**53**	7	**75**	36	5		16	20	5		12		**50**		**80**
**Anger**				11	7			5						**50**				

Note: ScG = Group with schizophrenia, ASD = Autism Spectrum Disorder w/o intellectual impairment, CG = Control group.

Participants with schizophrenia performed poorly (below the normative data) on the cognitive tests examining attention, mental processing speed, shifting ability, and verbal short-term memory (TMT-A = -.30, Standard deviation = 1.8; TMT-B = -1.3, Standard deviation = 1.8; 10-word list learning = -1.6, Standard deviation = 1.5). In order to explore whether the also poor performance of the group with schizophrenia on APT was affected by their limited cognitive abilities, we conducted Pearson’s correlations. Results revealed no association between APT scores and performance on TMT-A (r = .413, p = .126, z = .439), TMT-B (r = .360, p = .187, z = .377), and word list learning (r = .238, p = .393, z = .243).

Furthermore, we examined whether there was an association between APT performance for each of the two clinical groups and global functioning (group with schizophrenia = 52.4, standard deviation = 9.2; group with ASD w/o intellectual impairment = 80.3, standard deviation = 8.4). For the schizophrenia group an association was found between APT performance and GAF (r = .727, p = .002, z = 0.922). This was not the case for participants with ASD w/o intellectual impairment, however, since no association was found between their performance on the APT and GAF (r = .417, p = .122, z = .444).

### 3.2. Syntactic prosody task

Regarding syntactic prosody, groups’ performance on the fillers revealed a ceiling effect for all groups and no differences between them [filler subject-reading: χ^2^ = 1.024, p = .599, η^2^ = .024; filler object-reading: χ^2^ = 1.854, p = .396, η^2^ = .058] (see also Table 4). Similarly, no group differences were found on the recognition of prosody in the experimental items, either when they were in line with the subject [χ^2^ = 4.423, p = .110, η^2^ = .150], or the object reading [χ^2^ = 1.854, p = .396, η^2^ = .058] condition.

**Table 4:** Mean accuracy (standard deviations) in percentages for each group on the recognition of each condition of the syntactic prosody test.

	Experimental items (ambiguous)		Unambiguous fillers	
	Subject-reading	Object-reading	Subject- reading	Object-reading
ScG	80 (18)	93 (9)	99 (2)	100
ASD	68 (31)	89 (12)	99 (3)	99 (3)
CG	89 (9)	92 (7)	100	100

Note: ScG = Group with schizophrenia, ASD = Autism Spectrum Disorders w/o intellectual impairment, CG = Control group.

Further analysis using Mann-Whitney U tests demonstrated that patients with schizophrenia, although they performed worse compared to the control group on the subject-reading condition, this difference did not reach significance (U = 147.000, p = .150, r = .307). Similarly, no difference was found between the group with schizophrenia and the control group in the object reading condition of the experimental items (U = 100.000, p = .597, r = .111). The performance of the group with ASD w/o intellectual impairment, however, was significantly worse compared to the controls in the experimental items (U = 161.000, p = .044, r = .431). A closer look revealed that this difference was due to their performance in the subject reading condition, in which a marginal difference was found compared to the control group (U = 158.000, p = .055, r = .404). On the other hand, both the ASD w/o intellectual impairment and the control group performed similarly on the object reading condition (U = 137.500, p = .287, r = .222). All erroneous continuations of the participants of each group were in line with the opposite condition.

Pearson’s correlations revealed no association between participants’ with schizophrenia overall performance on the syntactic prosody task and their performance on TMT Part A (r = .373, p = .171, z = .391), TMT Part B (r = .404, p = .135, z = .428), and 10-word list learning (r = .415, p = .124, z = .441). As for the scores in GAF, it appears that they affected individuals’ with ASD w/o intellectual impairment performance (r = .573, p = .026, z = .652) on the syntactic prosody task, but not the group with schizophrenia (r = .442, p = .099, z = .474).

Lastly, we explored whether there is an association between each clinical group’s performance on the APT and the syntactic prosody task. No association was found for the group with schizophrenia (r = .330, p = .229, z = .343), but a significant positive correlation for the group with ASD w/o intellectual impairment (r = .632, p = .011, z = 0.745) was presented.

## 4. Discussion

The findings of the present study revealed important differences in the profile of adults with a recent diagnosis of schizophrenia and those with ASD w/o intellectual impairment with respect to their ability to recognize affective and syntactic prosody.

Regarding the APT performance, we found that individuals with schizophrenia had difficulty recognizing emotions across the board (with the exception of neutrality), but only marginally so on anger. The present findings are in agreement with previous studies in which deficits in affective prosody decoding were found in patients with schizophrenia [38–43]. Contrary, however, to Bozikas et al. [38], who used APT with adults with schizophrenia and found an impairment, in particular in the perception of negative emotions, such as anger and sadness, participants with schizophrenia in the present study revealed a more generalized deficit (except neutral prosody). Therefore, our hypothesis (a1) according to which participants with schizophrenia were expected to demonstrate difficulties with the recognition of negative emotions was not confirmed. The different outcomes may be attributed to differences in illness duration, as the patients of the present study had been diagnosed with schizophrenia less than 3 years prior to their participation, whereas for patients in Bozikas et al. [38] illness duration was much longer (mean = 10.76 years). The phase of schizophrenia has been found to affect patients’ facial emotion perception, as previous work has shown that patients with remitted schizophrenia perform better than patients with acute-phase schizophrenia (e.g., [61]). Therefore, differences in the phase of schizophrenia in the present, relative to other studies, may also account for impaired recognition of emotions via voice and differences in the findings of the present with other studies.

In contrast to the group with schizophrenia, the group with ASD w/o intellectual impairment demonstrated difficulty only in recognizing the emotion of surprise (see also [28]). Surprise can be considered as a more challenging emotion compared to others, as it can be accompanied by different feelings such as joy, anxiety, fear, or sadness [34,62]. Furthermore, surprise is believed to be less frequent in everyday life, compared to simpler emotions, such as happiness or sadness, and thus less straightforward to decipher [34]. If the aforementioned observation is valid for healthy adults, it is easy to see how surprise would be even less frequent in the everyday life of individuals with ASD w/o intellectual impairment, whose need for routine leaves very little room for new conditions and surprises. Our samples appear to be fairly typical, as previous research has shown that individuals with ASD w/o intellectual impairment face difficulties in recognizing specific emotions and not a generalized impairment in prosody decoding (e.g., [24]). The perception of the emotion of surprise appeared to also create problems for the larger cohort of individuals, who had taken part in Martzoukou et al. [28]. Therefore, our assumption (a2) according to which participants with ASD w/o intellectual impairment would encounter difficulties with the recognition of surprise was confirmed.

As for erroneous answers, all three groups demonstrated a similar pattern in the recognition of sadness, fear, and happiness. In particular, all participants sometimes mistook the emotions of fear for that of sadness and vice versa. Hiou et al. [59] found the same pattern in healthy individuals with the use of APT and attributed this misconception to the shivering voice used by the actor in the expression of both emotions. Similarly, in all three groups, the emotion of happiness was sometimes misperceived as indicating surprise. This confusion could be attributed to the fact that both emotions were expressed by the actor with high intensity. Of interest, however, is the observation that the opposite misconception did not occur (mistaking surprise for happiness) in either group. In most cases, both the controls and the participants with schizophrenia understood surprise as indicating neutrality, whereas participants with ASD w/o intellectual impairment mistook it as expressing fear. Although both answers can be explained by the fact that surprise can express both negative and positive feelings, it is interesting that controls and participants with schizophrenia revealed a similar pattern, whereas individuals with ASD w/o intellectual impairment performed in a different way. For anger and neutrality, no clear assumptions can be made since the erroneous answers were limited.

Regarding the syntactic prosody task, groups’ performance on the fillers revealed a ceiling effect for all groups, demonstrating that all participants were capable of completing the task and that the availability of morphosyntactic information aided their accurate processing of the sentences. Individuals with schizophrenia, although they performed poorer compared to controls on the subject reading condition, this difference did not reach significance. Therefore, it appears that the group with schizophrenia had no significant difficulty in recognizing syntactic prosody in general. These results are in line with previous studies exploring the perception of syntactic prosody in patients with schizophrenia [46,47] and confirm our (b1) hypothesis. As for the groups’ better performance on the object reading relative to subject reading, it reflects general parsing preferences, and it has also been demonstrated in previous studies using the same items and procedure (e.g., [28,63]).

For the group with ASD w/o intellectual impairment, though, the difference between the subject and the object reading condition was more pronounced, since they performed significantly poorer compared to the controls in the subject reading condition. Their performance affirms our (b2) hypothesis, according to which individuals with ASD w/o intellectual impairment were expected to face difficulties with the recognition of the subject reading condition of the experimental items. It appears that the subject reading condition is more challenging for the Greek speakers, since they have the tendency to expect an object after an optionally transitive verb (object reading condition) and not the beginning of another clause (subject reading condition) [26,57]. Therefore, in order for participants to correctly comprehend the subject reading sentence onsets and to provide a relevant continuation, they had to impede their syntactic preference for the object reading and to carefully comprehend the prosodic cues. Obviously, this task was difficult for participants with ASD w/o intellectual impairment (for more information see also Martzoukou et al. [28]).

The correlations test revealed that participants’ with schizophrenia verbal attention, mental processing speed, shifting ability, and short-term memory, as evaluated by the corresponding tests, did not affect their performance in either the APT or the syntactic prosody task. Thus, our (c) hypothesis was not confirmed, and, consequently, none of these variables could account for the schizophrenia group’s processing difficulties with respect to affective prosody.

On the contrary, global functioning level appeared to be related to the performance on the APT of the group with schizophrenia and to the performance on the syntactic prosody task of the group with ASD w/o intellectual impairment. These findings confirm hypothesis (d). More specifically, it appears that psychological, social, and professional functioning is closely related to individuals’ ability to recognise prosody. In the present study this relation became evident in the type of prosody in which each group performed worse compared to the controls. An association between social functioning and affective prosody recognition has also been reported in previous studies, although different tests were used (e.g., [61]).

## 5. Conclusions

Therefore, taken together the outcomes of the present study, it appears that patients with schizophrenia and ASD w/o intellectual impairment have different weaknesses with respect to the recognition of prosody. It seems that individuals with a recent diagnosis of schizophrenia have a generalized difficulty in recognizing affective prosody in particular. This difficulty, however, probably reflects impairment in expressing and perceiving emotions, as it has been reported in previous studies exploring emotions via prosody or pictures, and not an impairment in comprehending prosody per se, since their performance on the syntactic prosody task was comparable to that of the control group. On the other hand, adults with ASD w/o intellectual impairments appear to be facing a core difficulty in prosody processing which becomes evident in the most challenging conditions, such as the subject reading in syntactic prosody and the emotion of surprise in the affective prosody. Thus, performance on prosodic tests can be considered as a supplemental indication, along with other clinical indices, to aid in the diagnostic process. In particular, generalized poor performance on affective prosody could be indicative of a recent manifestation of the impairment, as occurs in schizophrenia, whereas difficulty in specific aspects of affective and syntactic prosody could indicate a more chronic condition, such as ASD w/o intellectual impairment.

Of course, the relatively small number of participants in each group may limit the generalizability of the findings. Moreover, we should take into consideration the high variability in the results of the clinical groups, as it becomes evident from the high standard deviations (e.g., Tables 2 and 4). This finding is in line with the great heterogeneity which characterizes both schizophrenia and ASD w/o intellectual ability; namely, symptoms and performance on tasks can vary significantly among individuals. An additional limitation may be the lack of use of a standardized diagnostic scale consistently across participants. Therefore, clearly, more research is needed with a greater sample size.

## Supporting information

S1 File (XLSX)
